# Dexrazoxane-afforded protection against chronic anthracycline cardiotoxicity *in vivo*: effective rescue of cardiomyocytes from apoptotic cell death

**DOI:** 10.1038/sj.bjc.6605192

**Published:** 2009-07-21

**Authors:** O Popelová, M Štěrba, P Hašková, T Šimůnek, M Hroch, I Gunčová, P Nachtigal, M Adamcová, V Geršl, Y Mazurová

**Affiliations:** 1 Department of Pharmacology, Faculty of Medicine, Charles University in Prague, Šimkova 870, Hradec Králové 500 38, Czech Republic; 2 Department of Biochemical Sciences, Faculty of Pharmacy in Hradec Králové, Charles University in Prague, Heyrovského 1203, Hradec Králové 500 05, Czech Republic; 3 Department of Histology and Embryology, Faculty of Medicine, Charles University in Prague, Šimkova 870, Hradec Králové 500 38, Czech Republic; 4 Department of Biological and Medical Sciences, Faculty of Pharmacy in Hradec Králové, Charles University in Prague, Heyrovského 1203, Hradec Králové 500 05, Czech Republic; 5 Department of Physiology, Faculty of Medicine, Charles University in Prague, Šimkova 870, Hradec Králové 500 38, Czech Republic

**Keywords:** anthracyclines, cardiotoxicity, cardioprotection, dexrazoxane, apoptosis

## Abstract

**Background:**

Dexrazoxane (DEX, ICRF-187) is the only clinically approved cardioprotectant against anthracycline cardiotoxicity. It has been traditionally postulated to undergo hydrolysis to iron-chelating agent ADR-925 and to prevent anthracycline-induced oxidative stress, progressive cardiomyocyte degeneration and subsequent non-programmed cell death. However, the additional capability of DEX to protect cardiomyocytes from apoptosis has remained unsubstantiated under clinically relevant *in vivo* conditions.

**Methods:**

Chronic anthracycline cardiotoxicity was induced in rabbits by repeated daunorubicin (DAU) administrations (3 mg kg^−1^ weekly for 10 weeks). Cardiomyocyte apoptosis was evaluated using TUNEL (terminal deoxynucleotidyl transferase biotin-dUTP nick end labelling) assay and activities of caspases 3/7, 8, 9 and 12. Lipoperoxidation was assayed using HPLC determination of myocardial malondialdehyde and 4-hydroxynonenal immunodetection.

**Results:**

Dexrazoxane (60 mg kg^−1^) co-treatment was capable of overcoming DAU-induced mortality, left ventricular dysfunction, profound structural damage of the myocardium and release of cardiac troponin T and I to circulation. Moreover, for the first time, it has been shown that DEX affords significant and nearly complete cardioprotection against anthracycline-induced apoptosis *in vivo* and effectively suppresses the complex apoptotic signalling triggered by DAU. In individual animals, the severity of apoptotic parameters significantly correlated with cardiac function. However, this effective cardioprotection occurred without a significant decrease in anthracycline-induced lipoperoxidation.

**Conclusion:**

This study identifies inhibition of apoptosis as an important target for effective cardioprotection against chronic anthracycline cardiotoxicity and suggests that lipoperoxidation-independent mechanisms are involved in the cardioprotective action of DEX.

Anthracycline antibiotics (ANT, e.g., doxorubicin, daunorubicin (DAU) or epirubicin) rank among the most potent and clinically important anticancer drugs ever developed. However, their clinical utility is markedly hampered by a significant risk of cardiotoxicity, especially that of a chronic and delayed type (Jones *et al*, 2006). The current conceptual model proposes that each anthracycline dose (cycle) induces an amount of damage to the myocardium. This injury, however, may stay clinically silent as long as the cumulative damage to cardiomyocytes does not exceed a certain functional reserve of the myocardium (Ewer and Benjamin, 2006). With an increasing cumulative dose, alterations to myocyte ultrastructure may progress further towards end-stage degeneration and cell death (Billingham *et al*, 1978; Von Hoff *et al*, 1979). The ANT cardiomyopathy that develops in this manner manifests itself in severe heart failure with poor overall prognosis (Jones *et al*, 2006; Gianni *et al*, 2008).

Anthracycline cardiotoxicity has been traditionally associated with oxidative stress-induced injury with a catalytic involvement of free cellular iron (Keizer *et al*, 1990). For many years, it has been believed that reactive oxygen species (ROS)-induced degenerative changes and subsequent non-programmed cell death of the cardiomyocytes are the primary (or even exclusive) hallmarks of ANT cardiomyopathy (Billingham *et al*, 1978; Godfraind, 1984; Lewis and Silver, 2001). However, with the wider appreciation of the function of programmed cell death in cardiac diseases, this view is undergoing considerable change. More recently, several experimental *in vitro* as well as *in vivo* reports have suggested that the exposure of cardiomyocytes to ANTs also triggers a well-orchestrated apoptotic mode of cell death (Sawyer *et al*, 1999; Arola *et al*, 2000; Nakamura *et al*, 2000; Yamaoka *et al*, 2000; Kotamraju *et al*, 2004; Konorev *et al*, 2008).

Dexrazoxane (DEX, ICRF-187) is the only well-established and clinically approved cardioprotectant against ANT cardiotoxicity (Wouters *et al*, 2005). The DEX is traditionally characterised as a prodrug, which is activated inside cardiomyocytes to the metal-chelating metabolite ADR-925. The latter active form of the drug is proposed to prevent ANT-induced and ROS-mediated cellular degeneration and non-programmed death of cardiomyocytes (Van Vleet *et al*, 1980; Herman *et al*, 1981). However, an *in vitro* study using isolated cardiomyocytes suggested that, in clinically relevant concentrations of ANTs, DEX may instead work through the prevention of programmed (apoptotic) cell death (Sawyer *et al*, 1999).

Hence, the main aim of this study was to examine whether DEX-afforded cardioprotection against chronic ANT cardiotoxicity *in vivo* involves a rescue of cardiomyocytes from apoptosis. In addition, the effects of DEX on the main apoptotic pathways and relations to lipoperoxidation were investigated.

## Materials and methods

### Animals and study design

This study was conducted using the previously well-established and accepted model of chronic ANT cardiotoxicity in rabbits (Gersl and Hrdina, 1994; Simunek *et al*, 2004; Sterba *et al*, 2006). Chinchilla male rabbits (3.48±0.03 kg) were randomised into three groups receiving (1) saline (1 ml kg^−1^, *i.v., n*=8; Natrium Chloratum, Biotika, Slovenská, L'upča, Slovakia); (2) DAU (3 mg kg^−1^, *i.v*., *n*=11; Daunoblastina; Pharmacia Italia, Nerviano, Italy); and (3) DEX (60 mg kg^−1^, *i.p.*, *n*=8; Cardioxane, Novartis, Basel, Switzerland) 30 min before each DAU administration (DEX+DAU group). All the substances were administered once weekly for 10 weeks. Administration of drugs, blood sampling and non-invasive measurements during the study were carried out under combined anaesthesia – ketamine (50 mg kg^−1^, *i.m.*; Narketan, Vétoquinol AG, Ittigen, Switzerland) and midazolam (1.25 mg kg^−1^, *i.m.*; Midazolam Torrex, Torrex Chiesi Pharma, Vienna, Austria). Pentobarbital (30 mg kg^−1^, *i.v.*; Sigma-Aldrich, Prague, Czech Republic) was used for anaesthesia during final invasive haemodynamic measurements and for an overdose of animals at the end of the experiment.

The study was terminated 5–7 days after the last drug administration. During the subsequent autopsy, hearts were rapidly excised, washed and briefly perfused with ice-cold saline. Heart ventricles were transversely cut under the atrio-ventricular septum and a block of left ventricular (LV) tissue was used for histological examination. The rest of the LV free wall was snap-frozen and homogenised under liquid nitrogen and kept frozen at −80°C for further analysis.

All experiments were approved and supervised by the Ethical Committee of Charles University in Prague, the Faculty of Medicine in Hradec Králové, and were in accordance with the Institute of Laboratory Animal Resources (1996).

### Cardiac function measurements

During the time course of the experiment, LV systolic function was followed by echocardiography (Vivid 4, with a 10 MHz probe; GE Medical Systems Ultrasound; GE Healthcare, Chalfont, St Giles, UK). The LV fractional shortening (FS) was obtained from LV end-systolic and end-diastolic diameters determined by guided M-mode scanning from the left parasternal long axis view.

At the end of the study, an invasive examination of LV contractility was carried out using a Micro-Tip pressure catheter (2.3F, Millar Instruments, Houston, TX, USA) connected to a data acquisition system (Powerlab, ADInstruments Pty., Castle Hill, Australia). The Chart 5.4.2 software (ADInstruments Pty.) was used for data analysis and calculation of the first derivative of the LV pressure rise in the isovolumic phase of systole (index d*P*/d*t*_max_).

### Cardiac troponin determination

Plasma concentrations of cardiac troponins T and I were determined using an Elecsys Troponin T STAT Immunoassay (Roche Diagnostics, Basel, Switzerland) and an ADV AxSYM troponin I Immunoassay (Abbott Laboratories, East Windsor, NJ, USA).

### Histology and TUNEL labelling

Tissue blocks (∼3 mm thick) of the transversely sectioned LVs were fixed by immersion in 4% neutral formaldehyde for 3 days. Thereafter, they were embedded in paraffin and numbered serial sections (6 *μ*m thick) were cut. The first two sections in each set were stained with H&E and Masson's blue trichrome for morphological evaluation, whereas the third, sixth and ninth sections were used for a terminal deoxynucleotidyl transferase biotin-dUTP nick end labelling (TUNEL) assay using an *In situ* Cell Death Detection Kit AP (Roche Diagnostics), according to the manufacturer's recommendation. The representative photomicrographs were obtained with a MicroImage software version 4.51 (Media Cybernetics, Laboratory Imaging, Prague, Czech Republic).

### Quantification of TUNEL-positive nuclei

Three serial LV sections were taken from each heart (representing a distance of 42 *μ*m) and labelled with the TUNEL assay. Photomicrographs of the tissue ( × 200 magnification) were made in numbered series using an Olympus (Central Valley, PA, USA) AX 70 microscope equipped with a digital camera Pixelink PL-A642 (Vitana Corp., Ottawa, Ontario, Canada). Quantitative analysis was performed by NIS image-analysis software (Laboratory Imaging) using 20 randomly chosen fields that represented approximately 3.7 mm^2^ of the LV area on a given section. The selection of fields was based on the generation of random numbers by SigmaStat software (SPSS Inc., Chicago, IL, USA). Only the appropriately stained nuclei of the cardiomyocytes at the section level were carefully counted. The average number of TUNEL-positive nuclei per square millimetre of the LV tissue was determined.

### Caspases activity determination

The activities of individual caspases were determined in the LV myocardium using commercially available kits. Myocardial samples were homogenised in a Lysis Buffer (BioVision, Mountain View, CA, USA) on ice. After centrifugation, the supernatants were collected for a further analysis of caspase activity. Caspase 12 activity was determined using a Caspase-12 Fluorometric Assay Kit (BioVision), whereas the activities of caspase 3/7, 8 and 9 were determined using luminescence Caspase-Glo Assays (Promega, Madison, WI, USA) according to the manufacturer's instruction. The fluorescence and luminescence units were normalised on the protein content in each sample. The fold-increase in caspase activity was determined by a comparison of these results, with the levels determined in the samples obtained from control animals.

### Myocardial lipoperoxidation analyses

For a reliable analytical determination of malondialdehyde (MDA) in myocardial samples, a thiobarbituric acid-independent HPLC method was used. This method has been appropriately validated with respect to selectivity, precision, accuracy and linearity following standards of FDA Guidelines for Bioanalytical Method Validation (FDA Guidance Document 2001). Radioimmunoprecipitation assay buffer (500 *μ*l) was added to the LV samples (∼70 mg of tissue) and the mixture was homogenised and vortexed. After centrifugation, the supernatant was withdrawn and analysed according to Pilz *et al* (2000). Briefly, 50 *μ*l of NaOH (6 mol l^−1^) was added, and after vortexing, the solution was kept at 60°C for 30 min. The samples were then cooled on ice and 125 *μ*l of perchloric acid (35% (v/v)) was added. After centrifugation, 250 *μ*l of supernatant was taken and derivatisation was performed using 25 *μ*l of 5 mmol l^−1^ 2,4-dinitrophenylhydrazine. After 10 min in the dark, the solution was analysed using an HPLC system (Shimadzu, Kyoto, Japan).

In addition, the immunohistochemical detection of 4-hydroxynonenal (4-HNE) was used as a complementary approach to myocardial lipoperoxidation assessment on transverse sections of the LV myocardium (two per animal). Incubation with a monoclonal 4-HNE antibody (Oxis International Inc., Beverly Hills, CA, USA; dilution 1 : 40) was performed overnight at 4°C. Secondary biotinylated donkey anti-mouse antibody (Jackson ImmunoResearch Laboratories Inc., West Grove, PA, USA; dilution 1 : 500) was applied for 40 min at room temperature. For visualisation 3,3′-diaminobenzidine-tetrahydrochloride (Sigma-Aldrich) was used. Sections were counterstained with Gill's haematoxylin.

### Protein concentration determination

Protein concentrations in the analysed samples were determined using a BCA Assay Kit according to the manufacturer's protocol (Sigma-Aldrich).

### Data analysis

The statistical softwares, SigmaStat 3.5 (SPSS Inc.) and STATISTICA Cz (StatSoft, Tulsa, OK, USA), were used in this study. All data are expressed as mean±s.e.m. Statistical significance was determined using one-way ANOVA unpaired test or paired *t*-test. Correlation analyses were performed using Spearman's method and regression analysis. Two independent exploratory multivariate statistical methods (Hierarchical Tree Clustering and Principal Component Analysis) were used to identify the natural groupings of individual animals in this study with respect to the following evaluated parameters – LV cardiac function parameters, cardiac troponins and markers of apoptosis and lipoperoxidation (comprising a total of 10 variables).

## Results

### General toxicity

The general appearance and mortality of animals were recorded during the time course of the study. In the control group, no premature mortality was observed and body weights were significantly increased during the experiment as compared with the initial values (3.50±0.03 *vs* 4.64±0.13 kg; *P*<0.01). On the other hand, DAU treatment resulted in premature death in two out of eleven animals (18%) and no change in body weight was observed in this group (3.45±0.07 and 3.47±0.23 kg, beginning *vs* end; NS). Furthermore, in prematurely dead animals, a necropsy examination revealed marked signs of blood congestion involving massive hydrothorax (∼60 ml) in both animals, in addition to ascites (25 and 35 ml). In addition, in comparison with the control group, the heart-to-body-weight ratio was found to be significantly increased in this group (2.82±0.18 *vs* 2.15±0.10 g kg^−1^, respectively, *P*<0.05).

In contrast to the DAU group, all DEX co-treated animals survived until the end of the study and their well-being was evidenced by a significant body weight increase during the experiment (3.50±0.07 and 3.99±0.07 kg, beginning *vs* end; *P*<0.01). No hydrothorax or ascites were found in this group and also the heart-to-body-weight ratio (2.55±0.13 g kg^−1^) showed no significant difference compared with that in the control group.

### Left ventricular systolic function

**Figure 1:**
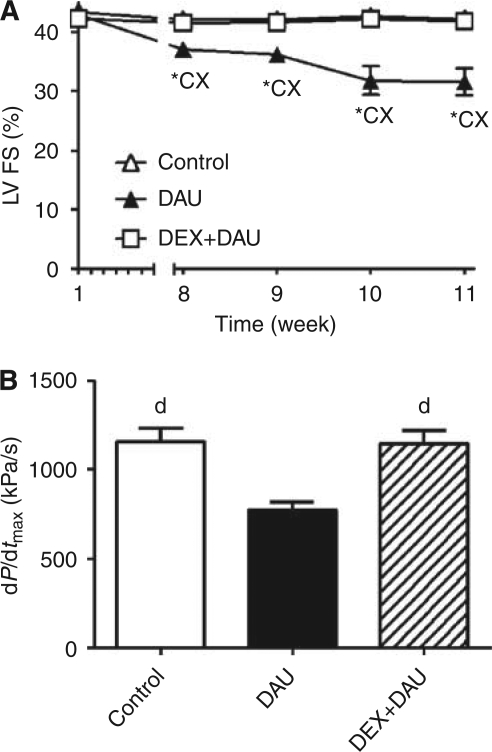
(**A**) Echocardiographically determined left ventricular (LV) fractional shortening (FS) during the time course of experiment. (**B**) Invasively determined index of LV contractility (index d*P*/d*t*_max_) at the end of the experiment. Statistical significance in comparison with ‘^*^’ the initial values within each group (paired *t*-test, P<0.05), ‘c’ control, ‘d’ daunorubicin and ‘x’ DEX+DAU groups (ANOVA, *P*<0.05).

Echocardiographically determined FS revealed a progressive and significant decline in LV systolic function in DAU-treated animals, starting by the eighth week (Figure 1A). In addition, an impaired LV systolic performance was also found in this group by invasive contractility examination performed at the end of the study (Figure 1B). In contrast, animals receiving DEX before each DAU injection showed nearly the same systolic function as the controls, which was evident from the outcomes of both types of the LV systolic examination (Figure 1A and B).

### Plasma concentrations of cardiac troponins

**Figure 2:**
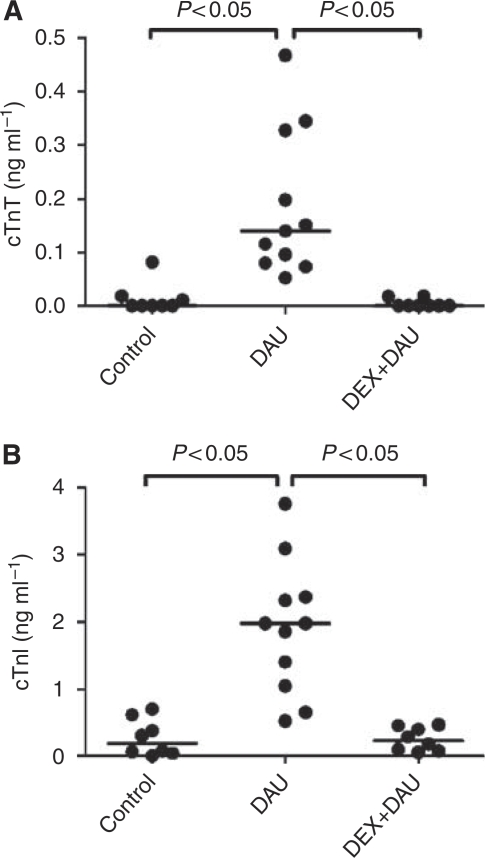
(**A**) Plasma concentrations of cardiac troponin T (cTnT) and (**B**) troponin I (cTnI) in individual animals at the end of the experiment.

Repeated administration of DAU led to significant elevations of plasma levels of troponins T and I. Correspondingly, DEX-afforded cardioprotection was associated with significantly lower plasma levels of both cardiac biomarkers (Figure 2A and B).

### Histological examination

**Figure 3:**
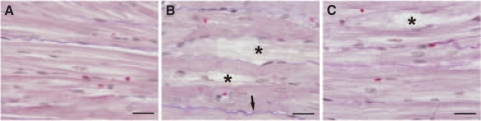
(**A**–**C**) Histological examination of the left ventricular (LV) myocardium. Masson's blue trichrome; bar=20 *μ*m. In the control group (**A**), normal structure of the myocardium was observed. In the DAU group (**B**), foci of cardiomyocytes showing profound degeneration (asterisks) were found. Marked morphological changes comprise cytoplasmic vacuolisation, cell swelling and loss of myofibrils resulting in cell death with subsequent development of interstitial fibrosis (arrow). The LV myocardium of DEX co-treated animals (**C**) showed largely preserved normal morphology with only subtle changes; signs of cardiomyocyte degeneration were scarce (asterisk).

A light microscopy examination of the LV myocardium of rabbits receiving DAU revealed profound focal toxic damage (Figure 3B). These changes comprised cellular degeneration with a mild-to-prominent loss of myofibrils and cytoplasmic vacuolisation. The advanced degenerative changes resulted in cell death of the non-programmed type. The affected cardiomyocytes were observed to undergo cellular swelling and gradual disintegration. Furthermore, whenever the dead cells formed larger foci, their remnants were replaced by a subsequent proliferation of fibrotic tissue that resulted in interstitial fibrosis. In sharp contrast, an examination of samples obtained from both the control and the DEX+DAU groups revealed a comparable morphological picture, with only subtle differences observed (Figure 3A and C). Importantly, the degenerative changes resulting in cell death, conspicuous in the DAU group, were rarely seen in the DEX+DAU group.

### TUNEL labelling and quantification of TUNEL-positive nuclei

**Figure 4:**
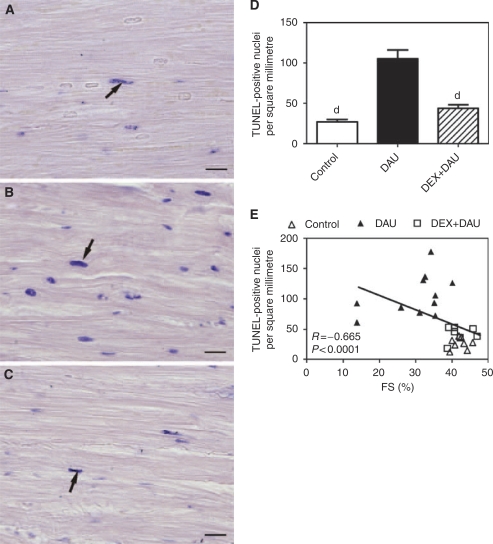
Terminal deoxynucleotidyl transferase biotin-dUTP nick end labelling (TUNEL) assay. (**A**–**C**) Representative samples of left ventricular (LV) myocardium labelled with TUNEL in the control (**A**), DAU (**B**) and DEX+DAU (**C**) groups. The arrows indicate examples of TUNEL-positive nuclei; bar=20 *μ*m. (**D**) Quantitative analysis of TUNEL assay. Statistical significance (ANOVA, *P*<0.05) in comparison with ‘d’ daunorubicin group. (**E**) Scatterplot of the LV fractional shortening *vs* number of TUNEL-positive nuclei per square millimetre and results of the Spearman's correlation analyses.

Examination of the TUNEL-labelled LV tissue sections showed only few positive cells in the investigated areas within the control group (Figure 4A). However, nuclear TUNEL positivity was significantly increased in the myocardium of DAU-treated animals (Figure 4B and D). The majority of TUNEL-positive nuclei were found in the cardiomyocytes that lacked any signs of typical degenerative changes, or these changes were present in their initial stages only. Unlike degenerative changes, TUNEL positivity did not show any regular pattern of appearance in the LV myocardium.

In the DEX+DAU group, the number of TUNEL-positive nuclei significantly declined to nearly the same values as determined in the control animals (Figure 4C). TUNEL-positive nuclei were found only in scattered isolated cells without any specific pattern. In addition, in individual animals, this parameter showed a significant correlation with LV systolic function (Figure 4E).

### Caspase activity

**Figure 5:**
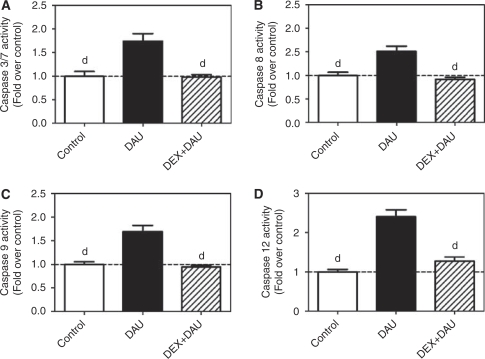
(**A**–**D**) Caspase activities in the left ventricular (LV) myocardium: caspase 3/7 (**A**), caspase 8 (**B**), caspase 9 (**C**) and caspase 12 (**D**). Statistical significances (ANOVA, *P*<0.05) in comparison with ‘d’ daunorubicin group.

**Figure 6:**
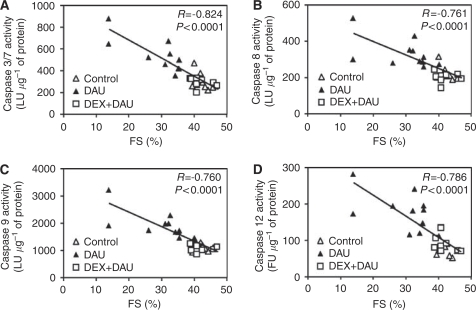
(**A**–**D**) Scatterplots of the cardiac function (LV fractional shortening (FS)) *vs* activity of individual caspases as determined by Spearman's correlation analyses: caspases 3/7 (**A**), caspase 8 (**B**), caspase 9 (**C**) and caspase 12 (**D**). LU, luminescence units; FU, fluorescence units.

Determination of the activity of all major caspases associated with apoptotic signalling showed triggering of multiple apoptotic pathways in the LV myocardium of DAU-treated animals. As seen in Figure 5A, chronic ANT treatment resulted in a significant increase in the activity of the executive downstream caspases 3 and 7, in comparison with that in the control group. In addition, the activities of caspases 8, 9 and 12 were also found to be increased in the DAU group (Figure 5B–D). In the DEX co-treated animals, the activity of the executive caspases 3/7 was significantly lower than that in the DAU-alone group (Figure 5A). Furthermore, activation of all major upstream pathways (i.e., caspases 8, 9 and 12) was effectively prevented by DEX co-treatment. There were no significant differences in the activities of individual caspases between the control and the DEX+DAU group (Figure 5B–D). Interestingly, the activities of these caspases showed a significant and strong correlation with LV systolic function (Figure 6A–D).

### Myocardial lipoperoxidation analyses

**Figure 7:**
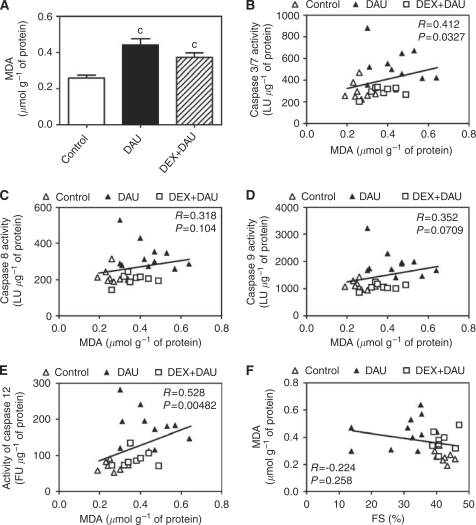
(**A**) Total malondialdehyde (MDA, marker of lipoperoxidation) levels in LV myocardium. Statistical significances (ANOVA, *P*<0.05) in comparison with ‘c’ control group. (**B**–**E**) Scatterplots of MDA levels in the left ventricular myocardium *vs* activity of individual caspases and results of the Spearman's correlation analyses: caspases 3/7 (**B**), caspase 8 (**C**), caspase 9 (**D**) and caspase 12 (**E**). (**F**) Scatterplot of myocardial MDA levels *vs* LV fractional shortening (FS) and results of the Spearman's correlation analyses. LU, luminescence units; FU, fluorescence units.

Using a selective HPLC method, significantly increased levels of total MDA(a widely used marker of lipoperoxidation) were determined in the LV myocardium of DAU-treated animals (Figure 7A). Surprisingly, DEX co-administration was not associated with a significant decrease in MDA levels. The MDA levels in the DEX+DAU group remained significantly higher than those in the controls and were close to the levels determined in the DAU-alone group. Furthermore, the MDA levels showed only a poor correlation with caspase 3 activity (Figure 7B) and no significant correlation with the activities of caspases 8 and 9 (Figure 7C and D). Interestingly, no association was also found between myocardial MDA levels and LV systolic function (Figure 7F).

**Figure 8:**
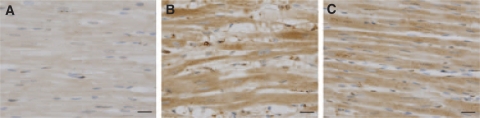
Representative samples of left ventricular (LV) myocardium showing 4-hydroxynonenal staining in the control (**A**), DAU (**B**) and DEX+DAU (**C**) groups.

The immunohistochemical detection of 4-HNE (another independent marker of lipoperoxidation) in the LV myocardium revealed a markedly higher response in the samples obtained from DAU-treated animals than in corresponding controls (Figure 8A and B). The 4-HNE signal was predominantly detected within the cardiomyocytes, whereas interstitial cells showed poor immunoreactivity. The myocardium of DEX co-treated animals (Figure 8C) showed a 4-HNE signal intensity that was largely comparable with that in the DAU-alone group. Although in some cases a moderate degree of reduction of the 4-HNE signal could be found in the DEX+DAU group, it was clearly always very far from being complete. No distinct changes in 4-HNE signal localisation could be found between the DAU and DEX+DAU groups.

### Exploratory data analysis

**Figure 9:**
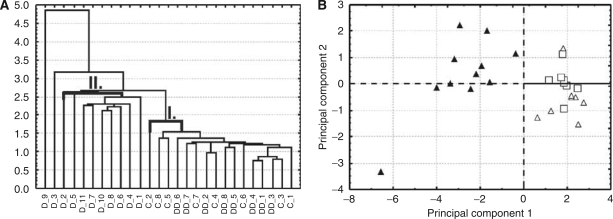
(**A**) Horizontal Hierarchical Tree Plot. The analysis comprised LV functional parameters (FS and d*P*/d*t*_max_), the plasma levels of cardiac troponins T and I, markers of apoptosis (TUNEL positivity and activities of caspases) and lipoperoxidation (nine variables) in all experimental groups. The analysis resulted in two main clusters. The first cluster covers exclusively the individuals from the control (C) and DEX co-treated group (DD), whereas the second cluster contains only the animals from the daunorubicin group (D), where almost all animals appeared in one cluster. (**B**) Principal Component Analysis (PCA) scatterplots. This analysis of the same variables also revealed two distinctly separated clusters comprising the DEX+DAU and control groups in the first, with all animals from the DAU group in the second cluster. Treatment groups: control (▵), DAU (▴) and DEX+DAU (□).

Hierarchical Tree Clustering (Figure 9A) and Principal Component Analysis (Figure 9B) were used to identify natural groupings within the 27 individual animals in this study with respect to the index of their LV functions (FS and d*P*/d*t*_max_), the plasma levels of cardiac troponins T and I, markers of apoptosis (TUNEL positivity and activities of caspases) and lipoperoxidation. Both exploratory data analysis approaches independently resulted in the grouping of all examined animals into two well-separated clusters. In the first cluster (I), all animals from the control and the DEX+DAU groups were found, whereas the individuals from the DAU group were arranged into a second cluster (II). Almost all animals from the DAU group appeared in one cluster, with the exception of two animals (prematurely dead) associated with the most profound changes. In addition, this exploratory data analysis identified caspase activities as the parameters with the highest impact on the natural grouping of animals.

## Discussion

In this study, repeated chronic DAU administration resulted in increased mortality, which was accompanied by severe blood congestion and heart failure. Echocardiographically determined FS revealed a progressive decline in LV systolic function, and this was further supported by an independent LV catheterisation examination performed at the end of the experiment. Dexrazoxane (ICRF-187), administered before each DAU injection, was capable of fully protecting the animals from both premature death and LV dysfunction. These findings illustrate the excellent cardioprotective potential of DEX, and the overall result is well in line with the outcomes of preclinical studies and clinical trials (Cvetkovic and Scott, 2005).

Histological examination of the LV myocardium in the DAU-treated animals revealed profound focal damage. These advanced degenerative changes (mainly cytoplasmic vacuolisation and loss of myofibrils) evidently resulted in non-programmed cell death. Such cardiomyocytes showed cellular swelling and a gradual disintegration. Importantly, DEX afforded very effective cardioprotection against these degenerative changes in a degree evidently comparable with previously published reports using morphometric quantitation (Herman *et al*, 1981, 1985, 1994). At the same time, the plasma levels of both cardiac troponins T and I were found to be markedly increased. The troponin concentrations found in this study were indeed lower than those associated with acute ischemia–reperfusion injury or isoproterenol cardiotoxicity (Bertinchant *et al*, 2000). However, it should be taken into account that chronic anthracycline cardiotoxicity progresses very slowly (several weeks) and is typically of a focal nature. Although the precise mechanism of troponin release into the blood stream has not yet been revealed, traditionally it is linked to necrotic death of cardiomyocytes, when the integrity of the cytoplasmic membrane is compromised (Horenstein *et al*, 2000; Wallace *et al*, 2004; Arbustini *et al*, 2008). Hence, DEX administration provided nearly a full protection of myocytes from the above-described advanced degenerative changes, as well as from the rise of cardiac troponin plasma levels. Specifically, the above-stated processes suggest a rescue of cardiomyocytes from non-programmed cell death, which likely triggers the process of cardiac remodelling. These findings correspond well with the widely accepted concept of DEX-afforded cardioprotection (Van Vleet *et al*, 1980; Herman *et al*, 1981).

In addition to the ‘traditional’ pathway of ANT-induced and ROS-mediated cardiomyocyte degeneration possibly resulting in non-programmed cell death, several lines of evidence have shown that apoptotic cell death may also be involved in anthracycline cardiotoxicity (Sawyer *et al*, 1999; Arola *et al*, 2000; Nakamura *et al*, 2000; Yamaoka *et al*, 2000). This is in contrast to very early reports (Zhang *et al*, 1996). In good agreement with numerous recent reports, we have shown that chronic ANT treatment leads to a significant increase in the number of TUNEL-positive nuclei in the LV myocardium, along with an increased activity of the executive downstream caspases 3 and 7. Interestingly, both these parameters corresponded well with the LV systolic function in individual animals. Previous studies have suggested in the main that either the intrinsic (i.e., mitochondrial, caspase 9 dependent) (Childs *et al*, 2002) or extrinsic (receptor mediated, caspase 8dependent) (Nakamura *et al*, 2000) pathways are pivotal in the ANT-induced triggering of cardiomyocyte apoptosis. Our investigation showed the activation of both caspases 8 and 9, thereby suggesting a concomitant involvement of both previously reported pathways and/or a cross talk between them. In addition, we have shown for the first time that the recently described endoplasmic/sarcoplasmic reticulum (ER/SR) pathway (caspase 12-mediated) might also contribute to chronic ANT cardiotoxicity. So far, this pathway has been reported only at the *in vitro* level (Kim *et al*, 2006), or as a result of the administration of a single high (supratherapeutic) dose of doxorubicin *in vivo* (Jang *et al*, 2004). Our present findings agree well with the available data, as profound ultrastructural and functional effects on ER/SR have been reported after repeated administration of ANTs *in vivo* (Minotti *et al*, 2004).

It is noteworthy that, in this study, we have observed that signs of significant cell degeneration and nuclear TUNEL positivity were only very rarely present in a single cardiomyocyte. Instead, although both these processes evidently co-existed in the LV myocardium as a whole, they typically affected different cardiomyocytes. We failed to find any regular pattern of distribution of cells with TUNEL-positive nuclei within the LV myocardium. Generally, apoptosis seemed to have a less significant function within the foci of the cardiomyocytes exhibiting advanced degenerative changes. Although advanced degenerative changes seemed to be more prominent in the animals with the most profound cardiotoxicity, we cannot yet apportion the contribution of each of these processes to heart failure induced by anthracyclines. At this point, it should be stressed that, although apoptosis is completely finished within several hours, degenerative changes evidently progress relatively slowly, which hinders an appropriate head-to-head comparison of the real contribution of both processes to the clinical manifestation of ANT cardiotoxicity.

Although there is solid evidence for induction of apoptosis after the exposure of cardiac myocytes to ANTs, one of the important questions has remained hitherto unanswered: Is modulation of apoptotic cell death involved in the remarkably effective cardioprotection afforded by DEX? Scanty data from the literature have suggested that this might be true, at least at the level of the isolated cardiomyocytes exposed acutely to ANTs (Sawyer *et al*, 1999). Nevertheless, in this study, we showed for the first time that DEX is able to significantly and nearly completely rescue cardiomyocytes from ANT-induced apoptosis under clinically relevant chronic *in vivo* conditions. Furthermore, the current investigation reveals that DEX prevents the ANT-induced activation of all major upstream (i.e., caspases 8-, 9- and 12 dependent) pathways. Importantly, all the examined parameters of apoptosis showed a significant and strong correlation with LV systolic function in individual animals. Indeed, the ability of DEX to prevent the triggering of multiple apoptotic pathways may account for its high efficacy in the prevention of ANT-induced apoptotic cell death in the LV myocardium. Further information regarding the relationships between the apoptotic and other studied parameters was obtained from two independent exploratory data analyses. These revealed that DEX showed a clear tendency towards reduction of both apoptotic and non-apoptotic cell death. It is noteworthy that the activities of apoptotic caspases were determined to have the strongest impact on the natural grouping of individual animals, which highlights apoptosis as an extremely important target for cardioprotective intervention with DEX. However, it should be stated that we cannot draw any definitive conclusion regarding the relative importance of these cardioprotective pathways at this stage.

Although our results convincingly show that DEX rescues cardiac myocytes from programmed and non-programmed cell death, the mechanisms responsible for these effects remain elusive. So far, the cardioprotective effects of DEX have been mostly attributed to its ring-opening hydrolysis product ADR-925 (Kwok and Richardson, 2000; Cvetkovic and Scott, 2005). This compound has been shown to displace iron from ANT–iron complexes, and hence it has been assumed to prevent redox cycling and production of extremely toxic hydroxyl radicals (Hasinoff *et al*, 1998). Nevertheless, using the same experimental model, the degree of cardioprotection afforded by DEX in this study was apparently much higher than that obtained previously with much stronger and selective aroylhydrazone iron chelators (Simunek *et al*, 2005b; Sterba *et al*, 2006, 2007). Furthermore, deferiprone, a novel clinically used cell permeable iron chelator, failed to afford any meaningful cardioprotection under identical conditions (Popelova *et al*, 2008). Hence, these data imply that the iron-chelating properties of a compound are not the main determinants of its cardioprotective action.

The ANT-induced formation of ROS is believed to induce a profound myocardial oxidative injury to most of the biomolecules in the LV myocardium and to phospholipids in particular (Keizer *et al*, 1990). Hence, we sought to determine whether DEX-afforded cardioprotection involves the prevention of ROS-induced damage as a common denominator by means of total MDA measurement by HPLC in whole-tissue extract prepared from LV myocardium. This analytical approach is free of the known drawbacks associated with the older thiobarbituric acid-based methods (Janero, 1990; Esterbauer *et al*, 1991; Pilz *et al*, 2000). Using this method, we have found significantly increased levels of MDA in the LV myocardium of DAU-treated animals, suggesting that oxidative damage was associated with the treatment. Although these data are well in line with literature and traditional concepts (Doroshow *et al*, 1980; Sarvazyan, 1996; Zhou *et al*, 2001), the finding that DEX failed to overcome ANT-induced lipoperoxidation was highly novel and rather surprising. It is noteworthy that the analytical method used herein has been previously shown to be capable of documenting a reduction of lipoperoxidation provided by pharmacological cardioprotection using iron-chelating agents (Simunek *et al*, 2007). Moreover, similar results were also obtained using complementary immunohistochemical detection of 4-HNE as another marker of lipoperoxidation. These observations strikingly contrasted with the powerful cardioprotection afforded by DEX in these animals. In addition, the MDA levels showed only a poor agreement with caspase 3/7 activity, and no correlation with the activities of the main caspases of both extrinsic and intrinsic pathways (caspases 8 and 9) were found. Moreover, this marker of lipoperoxidation showed no relationship with LV systolic function. We cannot exclude, however, the fact that DEX-afforded protection against free radical injury may be primarily compartmentalised in origin, although the immunohistochemical analysis of 4-HNE did not suggest so. One also could expect that the protection afforded in any particular compartment should still be reflected in the total MDA levels, if in fact it is so crucial to the fate of the whole cell. Nevertheless, we did not observe any such trend in this study. These findings strongly suggest that the excellent cardioprotection obtained with DEX is not primarily dependent on the protection from ANT-induced lipoperoxidation. On the other hand, we cannot exclude that there is a compartment within the cell that might have a key function in ROS-dependent cardiotoxicity and DEX-afforded cardioprotection, although it does not have a striking impact on overall lipoperoxidation.

Using isolated rat cardiomyocytes, we have previously shown that the chelator, salicylaldehyde isonicotinoyl hydrazone (SIH), affords significant, although only partial, protection from DAU-induced cell death. However, this protective effect was not accompanied by any reduction of lipoperoxidation (Simunek *et al*, 2008). This finding contrasted with the results obtained with the same compound on the model of hydrogen peroxide-induced oxidative stress (Simunek *et al*, 2005a, 2007, 2008). In this latter case, the SIH-afforded protection of cardiomyocytes was markedly better and clearly oxidative stress dependent. Furthermore, in another study, DEX failed to mitigate doxorubicin-induced peroxidative damage to A549 human lung adenocarcinoma cells (Kaiserova *et al*, 2006).

Although further studies primarily designed to clarify this point deserve to be performed, at this stage, we come to the inevitable conclusion that the function of DEX has less to do with its ROS-reduction capability. This might provide us an explanation for the failure of previous attempts to provide effective cardioprotection against ANT cardiotoxicity using a whole range of antioxidants (e.g., vitamin E, acetylcysteine or flavonoids) in chronic experimental models (Van Vleet *et al*, 1980; Herman *et al*, 1985; Bruynzeel *et al*, 2007b) and randomised clinical trials (Dresdale *et al*, 1982; Legha *et al*, 1982; Myers *et al*, 1983; Bruynzeel *et al*, 2007a). From this point of view, our results further support the recent call for revisiting the classical ‘ROS and iron hypothesis’ of ANT cardiotoxicity and DEX cardioprotection (Gianni *et al*, 2008).

Indeed, the data from this study raise new questions about the actual mechanisms underlying the protective action of DEX against ANT cardiotoxicity. First, it should be noted that, unlike the iron-chelating compounds studied previously (Sterba *et al*, 2006, 2007; Popelova *et al*, 2008), the putative active metabolite of DEX (ADR-925) is far from being a selective iron chelator. Owing to this non-selectivity, it is plausible that intracellular chelation of other biologically important multivalent metal cations could participate in cardioprotective effects of DEX. Importantly, ANTs have been repeatedly shown to impair tightly regulated calcium homoeostasis in cardiac cells (Minotti *et al*, 2004; Wallace, 2007). We have previously shown that ANT-induced calcium overload can be prevented by DEX co-treatment (Simunek *et al*, 2005c). Furthermore, the determination of caspase 12 activity in this study suggested an involvement of ER/SR stress, which was preventable by DEX administration. In addition, others have reported that mitochondrial calcium overload and/or activation of calcium-dependent proteases are part of the key molecular mechanisms of ANT cardiotoxicity development (Solem *et al*, 1994; Lim *et al*, 2004).

Besides the above-discussed hypotheses that consider DEX solely as a prodrug of the metal-chelating agent ADR-925, some novel suggestions point at the importance of a direct cardioprotective involvement of DEX (Hasinoff and Herman, 2007; Lyu *et al*, 2007). The DEX is a known catalytic inhibitor of topoisomerase II and this action has also been considered as being responsible for its anticancer effects. The DEX binds directly to topoisomerase II and thereby locks the enzyme in a stable and closed clamp conformation around DNA (Roca *et al*, 1994). On the other hand, ANTs are also known to be topoisomerase II poisons that act through the stabilisation of topoisomerase II-DNA covalent complexes, resulting in DNA strand breaks (Minotti *et al*, 2004). It has been hypothesised that DEX, as a catalytic inhibitor, might have protective effects against topoisomerase II-poisoning agents (Lyu *et al*, 2007).

In addition, there are reports indicating that the beta isoform of topoisomerase II is abundant in the post-mitotic myocardium (Wang, 2002), including mitochondria that are prominently targeted by ANT cardiotoxicity (Wallace, 2003, 2007). A recent *in vitro* study conducted on a H9c2 rat cardiomyoblast cell line has suggested that DEX-promoted inhibition of topoisomerase II β may have a significant function in the cardioprotective action of the drug (Lyu *et al*, 2007). Another recent report dealing with a new DEX derivative (ICRF-161) has shown that, unlike iron-chelating properties, topoisomerase II inhibition might be of primary importance for effective cardioprotection under chronic *in vivo* conditions (Martin *et al*, 2009).

In conclusion, this study shows for the first time that DEX-afforded cardioprotection against chronic ANT cardiotoxicity *in vivo* is based not only on the rescue of cardiomyocytes from degenerative changes and non-programmed cell death but also on rescue from programmed cell suicide – apoptosis of the ventricular myocytes. ANT-induced apoptotic signalling seems to be very complex, but DEX was shown to block all the major apoptotic pathways. Clarification of the mechanisms responsible for the potent protective action of DEX against the apoptotic death of cardiac myocytes merits further study. Nevertheless, protection of cardiac myocytes does not seem to be primarily lipoperoxidation dependent. These findings point out to apoptosis being a part of the cardioprotective action of DEX, and further underline the need for revisiting the traditional but potentially oversimplified ‘ROS and iron hypothesis’ of ANT cardiotoxicity.
